# Paper-Based Resazurin Assay of Inhibitor-Treated Porcine Sperm

**DOI:** 10.3390/mi10080495

**Published:** 2019-07-25

**Authors:** Koji Matsuura, Wen-Hsin Wang, Alex Ching, Yu Chen, Chao-Min Cheng

**Affiliations:** 1 Department of Biomedical Engineering, Faculty of Engineering, Okayama University of Science, 1-1 Ridai-Cho Kita-Ku, Okayama 700-0005, Japan; 2 Institute of Biomedical Engineering, National Tsing Hua University, Hsinchu 30013, Taiwan; 3 St. Mark’s School of Texas, Dallas, TX 75230, USA; 4 Department of Urology, Chang Gung Memorial Hospital and Chang Gung University, Taoyuan 33302, Taiwan

**Keywords:** paper-based assay, porcine sperm motility, resazurin, redox reaction

## Abstract

Porcine sperm motility was assessed via resazurin reduction color change in sperm cells using a novel paper-based assay of our own design. We applied mixtures of resazurin solution and porcine semen onto hydrophilic test circles on our paper-based device and investigated the resulting reduction reaction expressed as red and blue color intensity (RBCI). We quantified this reaction using a blue/pink color ratio from our 8 × 3 = 24 bit RGB color image. To examine enzymatic reactivity in sperm cells, we used two inhibitors: 3-Nitropropanoic acid (3-NPA) and 3-Bromopyruvic acid (3-BP). 3-NPA inhibits the citric acid cycle and electron transfer reaction in mitochondria, but did not strongly reduce sperm motility in our tests. 3-BP decreases reactivity of both mitochondrial electron transfer and glycolytic enzymes in cytosol, which significantly lowers porcine sperm motility. RBCIs of 3-NPA- and 3-BP-treated samples were significantly lower compared to our untreated control (*p* < 0.025). Based on these results, we feel that resazurin can be used to estimate the amount of reductants with and without inhibitor treatment. For continued research assessing the molecular mechanisms of resazurin reduction in porcine sperm, a combination assay using two or more redox indicators (e.g., resazurin and Thiazolyl Blue Tetrazolium Bromide (MTT)) embedded into our paper-based device could further our understanding of sperm cell bioenergetics.

## 1. Introduction

**Figure 1:**
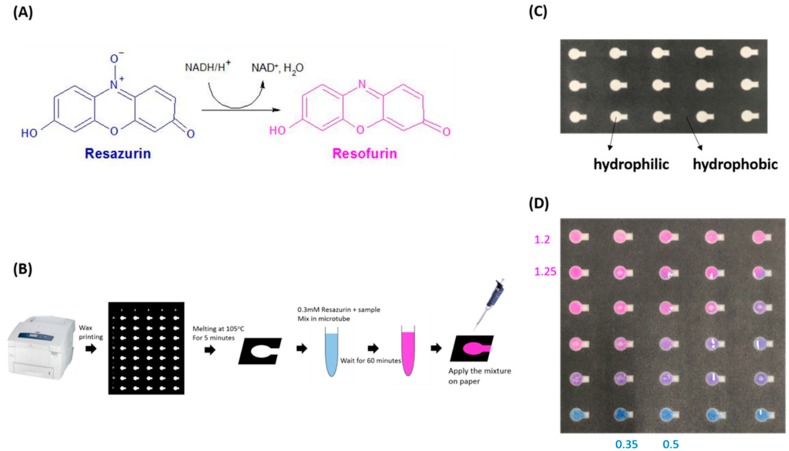
(**A**) Molecular structure of resazurin chemistry; (**B**) paper-based device preparation and resazurin assay methods; (**C**) the test pattern of the paper-based device; (**D**) a demonstration of color differences of the applied mixture of resazurin and semen on the paper-based device. Values in this figure are calculated red and blue color intensities (RBCIs) for test zones.

Resazurin (IUPAC name: 7-hydroxy-10-oxidophenoxazin-10-ium-3-one) is a redox indicator that irreversibly changes from its blue oxidized form to its pink reduced form resofurin. This change is also structural at a molecular level, as illustrated in Figure 1A. This redox reaction can be analyzed using optical absorption or fluorescence. Resazurin assays are used for natural water analysis [1], photocatalysis [2], disulfide oxidation analysis [3], measurement of ascorbate activity [4,5], and detection/determination of microbial cells [6], mammalian cell lines [7,8,9,10,11], and mammalian sperm cells [12,13,14,15,16,17,18,19]. Correlations between resazurin reduction within mammalian sperm and sperm parameters such as concentration, motility, or acrosomal integrity have been investigated [12,13,14,15,16,17,18,19]. The correlation between resazurin reduction and sperm motility is relatively lower than that between resazurin reduction and sperm concentration, suggesting that a redox reaction of resazurin can be used to detect reductants such as nicotinamide adenine dinucleotide (NADH) in sperm cells [11,14]. An enzymatic reaction assay using NADH in sperm would provide sperm characteristics, especially sperm motility, that could be more fully evaluated when used in combination with a resazurin assay and/or other assays. Sperm energetics analyses can provide important details on sperm motility, and redox-based assays can clarify sperm energetics mechanisms at the molecular level. 

Correlations between redox-based indicator color change, such as resazurin/Thiazolyl Blue Tetrazolium Bromide (MTT) reduction within mammalian sperm, and sperm parameters, such as viability and motility, have been investigated [15,16,18,20,21,22]. In previous research, MTT reduction rates in bovine, equine, and boar sperm samples correlated with viability using a LIVE/DEAD assay and eosin staining [20,21,22]. Photometer assays of resazurin reduction indicated that resazurin reduction correlated with motile boar sperm concentration [15,16]. However, Sabés-Alsina et al. concluded that no correlation between resazurin reduction and motility parameters were found in rabbit-sourced samples [18]. Higher mitochondrial activity in motile boar sperm indicated both higher mitochondrial transmembrane potential (ΔФ) and respiratory chain complex I activity [23]. In porcine sperm, electron transfer in mitochondria must be related to ΔФ, and decreasing ΔФ using inhibitors would contribute to decreased motility, which would in turn reduce the success rate of artificial insemination (AI).

A human sperm study of 3049 semen samples suggested that human sperm viability was associated with both motilities and DNA fragmentation, which are important for AI and in vitro fertilization (IVF) [24]. Ruiz-Pesini et al. investigated enzymatic activities of mitochondrial enzymes in the electron transfer system (complex I, II, III, and IV) of human sperm using a spectrophotometer and presented a relationship between the reactivity and percentages of progressive motile sperm [25]. Electron transfer reactions between these complexes would contribute to increased ΔФ and the production of adenosine triphosphate (ATP) to support sperm flagellum motion. The value of ΔФ is considered an indicator of sperm quality, sperm motility, and fertilization capacity [26,27,28,29,30]. Therefore, inhibition of electron transfer activities would contribute to decreased fertilization capacity of both porcine and human sperm. A redox reaction of resazurin can be potentially be used an indicator of electron transfer activities in sperm, because the reduction of resazurin is coupled with NADH oxidation, which is a key material of mitochondrial functions. 

Previous experiments were completed using photometric devices [15,16,18,20,21,22]. To provide precise, robust, portable, and inexpensive redox-based assay systems, paper-based assays have been developed for mammalian sperm using MTT [31,32,33,34]. We found a correlation between colorimetric results from a paper-based MTT assay and sperm motility as determined by inhibitor treatment, and suggested that the redox mechanism was related to flagellum beating [31,32]. Paper-based devices possess several advantages. Of particular note, they can be used to evaluate various indicators in a single assay, saving considerable time and cost over clinical methods requiring expensive tools such as a multi-plate reader (approximate cost, 4000 USD). 

Paper-based assay systems use smaller sample volumes, less costly materials, and less analytical equipment, especially in terms of optical equipment, compared to multi-plate, well-based systems. Typically, at least 20 μL of semen is required for conventional assay approaches. Our approach uses only 5 μL of semen. To use a plate reader to evaluate optical absorption, we would have to change optical systems to measure color intensity for each of our color indicators. This hinders the ability to complete rapid multi-color assays, something a paper-based system could complete with greater ease. Furthermore, a comparison of material and analytical tool costs would indicate that paper-based approaches are more easily undertaken, especially in resource-poor areas, and the resulting impact on human healthcare could, accordingly, be quite significant.

Borra et al. found a method for recording colorimetric resazurin assay results from a multi-well plate using a digital camera—a process that can also be applied to paper-based assays [10]. We need to evaluate the usability of resazurin in a paper-based assay system, and to consider critical redox reactions that might be leveraged to examine resazurin redox reactivity in response to enzyme inhibitors in sperm cells. We also need to optimize an assay protocol and design an optimal test pattern for resazurin reaction sperm analysis using a paper-based assay system. Reaction volume and resazurin concentration were adjusted to get a reliable color change using resazurin, and we mixed semen with resazurin solution in a microtube to facilitate uniform color change in our paper-based assay reaction zones. We choose the test pattern shown in Figure 1C for our paper-based resazurin assay because we wanted to calculate color intensity only from the circular reaction zone area, which would provide a uniformly colored region for calculations. When advances in this approach are complete and redox-based assays can be performed in a single experiment, sperm characteristics can be more completely evaluated based on molecular assay mechanisms.

Here, we investigated the effectiveness of a paper-based resazurin assay to evaluate porcine sperm parameters and the effects of inhibitors on enzymatic reaction activity in porcine sperm cells. 3-Nitropropanoic acid (3-NPA) is an inhibitor of both succinate dehydrogenase (SDH) in the citric acid cycle and mitochondrial electron transfer (complex II) [35,36,37,38]. 3-Bromopyruvic acid (3-BP) inhibits both mitochondrial electron transfer (complex II) and glycolysis [39]. We examined the effects of these inhibitors using a paper-based, colorimetric, resazurin assay.

## 2. Materials and Methods

### 2.1. Materials

Phosphate buffered saline (PBS) and 3-NPA were purchased from Sigma-Aldrich Chemical Co. (St. Louis, MO, USA). 3-BP was purchased from Alfa Aesar Co. (Ward Hill, MA, USA).

We prepared the MTT for coating our paper-based device test zones according to the research of Aziz et al. [20,21]. For this experiment, we printed our pattern onto chromatography paper (Grade 1 Qualitative Filter Papers, GE Healthcare Life Sciences, Little Chalfont, Bucks, UK), as shown in Figure 1B, using a wax-printer (Phaser 8560 wax printer, Xerox, Norwalk, CT, USA). After heating the paper with the patterned wax at 105 °C for 5 min, white hydrophilic and black hydrophobic regions formed on and through the chromatography paper, as shown in Figure 1C.

### 2.2. Porcine Sperm Preparation and Inhibitor Treatment Experiments

Porcine sperm was supplied by Artificial Insemination (AI) Center of Agricultural Technology Research-Institute Animal Technology Laboratories in Taiwan. Collection of semen was obtained one day before these experiments were performed. After confirming that sperm cells were qualified, they were placed into a Styrofoam box and maintained at a temperature of approximately 16 °C.

### 2.3. Sperm Motility and Resazurin Assays

Porcine semen was rewarmed to 37 °C in a water bath for 15 min. It was gently mixed before and after adding inhibitor agents, which were allowed to incubate with our sample for 30–90 min. We used iSperm (Aidmics Biotechnology, Taipei, Taiwan) to analyze inhibitor-treated samples as per our previous report [33]. Sperm concentration in the semen was from 500 to 1100 million sperm cells/mL. We mixed 200 µL of the semen with 200 µL of 0.3 mM aqueous resazurin solution in microtubes. After 60 min, we applied 5 µL aliquots of the mixture to several white reaction zones on our paper-based device. We checked the color on the paper and took a photo of the paper 1 min after mixture application. In inhibitor treatment experiments, we added 80 μL of 100 mM 3-NPA (solved in DW) or 8 μL of 100 mM 3-BP to 1 mL of the semen sample. The final inhibitor concentrations were 8 mM for 3-NPA and 0.8 mM for 3-BP, respectively. Following these inhibitor treatments, we examined sperm cell motility using iSperm.

We analyzed the resulting color intensity of the circular reaction zones on our paper-based assay using Image J (version 1.50, National Institutes of Health, Bethesda, MD, USA). Using red and blue color intensity (RBCI), we determined a value to represent the blue/pink color ratio based on our 8 × 3 = 24-bit RGB color image as follows (Equation (1)):

RBCI = (*RED* color intensity in the spot)/(*BLUE* color intensity in the spot) (1)

We calculated average red and blue color intensities in the five color spots used for one sample. Larger RBCI values (visually pink values) were indicative of resazurin having undergone greater reduction to resofurin, as shown in Figure 1D. We compared the average of 9–12 semen samples with and without inhibitor treatments.

### 2.4. Statistical Analysis

We used Student’s *t*-test to determine RBCI differences for sperm motilities. Usually, *p*-value after Bonferroni correction is calculated as α/n, where α is the significance level (0.05), and n is the number of pairs. Here, we compared two pairs (non-treated samples to 3-NPA-treated samples and non-treated samples to 3-BP-treated samples) among three groups, so that the value of n was equal to 2. We considered *p* < 0.025 = 0.05/2 to indicate a significant difference. The coefficient of determination (R^2^ value) for the relationship between porcine sperm motility and the resazurin color RBCI was calculated using Microsoft Excel (Microsoft Co. Ltd., Redmond, WA, USA) and the least-squares method.

## 3. Result

### 3.1. Resazurin Assay and Semen Parameters

**Figure 2:**
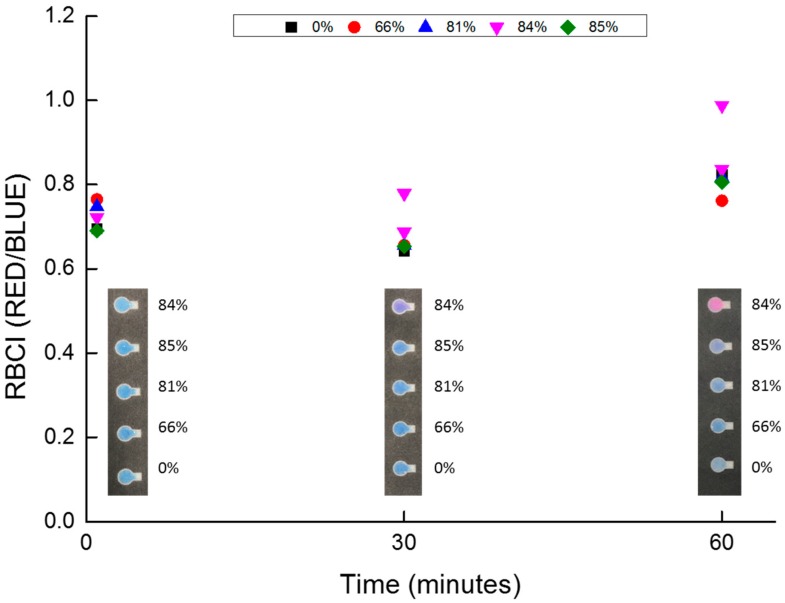
Relationship between RBCI and treatment time for the resazurin assay. Percentages in the square are motility of porcine sperm.

Figure 2 shows the relationship between treatment time after mixing and RBCI. We examined the RBCI differences during the reduction of resazurin. When we incubated semen with resazurin in microtubes for 1 min and 30 min, the redox reaction of resazurin did not finish, and the color change was not sufficient to evaluate RBCI. Although we did not collect any image data after the 60 min time point, the color change in the microtube appeared to completely finish at approximately 60 min, so we choose this as our reaction time standard.

Nicotinamide adenine dinucleotide phosphate (NADPH)/diaphorase/resazurin assay was used for analyses of isocitratedehydrogenase1 [40], and diaphorase is expressed in porcine sperms [41]. We concluded that the treatment time of 60 min was consistent with previous kinetic resazurin assay studies.

### 3.2. Inhibitor Treatment Effects and Inhibition Mechanism

**Figure 3:**
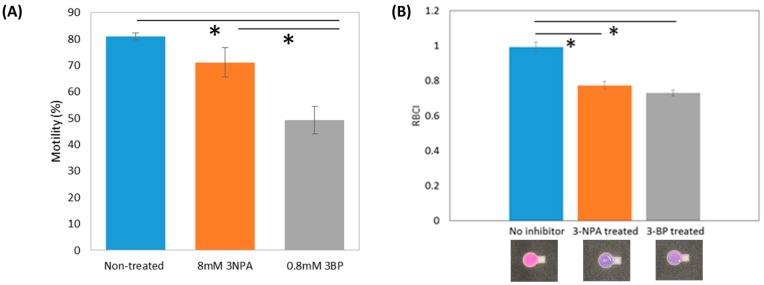
(**A**) Comparison of average motility for 60 min treatment without and with inhibitors (* *p* < 0.025); (**B**) comparison of average RBCI for 60 min treatment without and with inhibitors (* *p* < 0.025). Sample numbers of the groups without inhibitor, with 3-NPA treatment, and with 3-BP treatment were 12, 8, and 10, respectively. Error bars are standard errors (SE) of motility and average RBCIs.

Figure 3A suggests that 3-BP treatment induces a decrease in motility, as previously reported (*p* < 0.025) [33]. RBCI values for treated and untreated samples, as shown in Figure 3B, indicate that the average RBCI values for 3-NPA treated and 3-BP treated samples were significantly lower than that of the untreated samples (*p* < 0.025). Resazurin in untreated semen samples was strongly reduced by reductants within the sperm cells. The R^2^ value between porcine sperm motility and RBCI from our paper-based resazurin assay (0.265) was lower than that of our paper-based MTT assay (0.805), suggesting that the MTT assay was sensitive for sperm motility analysis [33].

## 4. Discussion

At a molecular level, the observed redox chemistry in porcine semen after 3-NPA and 3-BP treatments can be attributed to the fact that both inhibitors affect electron transfer activities to ubiquinone from FADH_2_ (Flavin adenine dinucleotide, hydroquinone form (complex II)). Moreover, 3-NPA inhibits SDH in the citric acid cycle, and Glyceraldehyde 3-phosphate dehydrogenase (GAPDH) activity during glycolysis is decreased by 3-BP treatment. Energy for mouse and porcine sperm motilities are mainly produced by glycolysis [33,42,43]. However, ATP from mitochondria also contributes to sperm motility [44,45,46,47]. Decreased RBCI following 3-NPA treatment corresponds to decreased concentration of reductants in porcine sperm by inhibition of the citric acid cycle and electron transfer. 3-BP inhibits both glycolytic enzyme and mitochondrial electron transfer. The RBCI of our 3-BP treated sample was similar to that of our 3-NPA treated sample. Based on our accumulated results, resazurin may be an indicator of reductants such as NADH and FADH_2_ [31,32,33].

There were limitations to this study, and further studies are warranted for greater understanding. Although we could evaluate resazurin color change following inhibitor treatment using a paper-based format, additional sperm characteristics beyond motility would provide additional valuable information that would advance testing approaches. We did not have the opportunity to evaluate data using total motile counts or other fertility parameters that would clarify and inform our colorimetric results by providing the number of motile sperm cells and motile sperm trajectory distributions, which are pursuits reserved for future study. It would be valuable to discuss the molecular mechanism of the porcine semen source resazurin color change by coupling the color change to indicators such as mitochondrial function, enzymatic activity analyses, and ΔФ detection. Research into the relationship between resazurin color change and mitochondrial enzymatic activities would further our understanding of redox indicator results for assaying sperm and their inter-relationship with motility mechanisms and sperm bioenergetics.

## 5. Conclusions

Resazurin color change on paper-based devices can be used for porcine sperm motility analyses. However, the correlation of resazurin color change by reduction to porcine sperm motility was weaker compared to the correlation of MTT reduction to sperm motility. This inhibition study suggests that resazurin can be used to analyze reductants in porcine sperm. A combination of MTT and paper-based resazurin assays may provide important information on energy metabolism related to sperm motility in sperm cells.
